# Kindergarten-based obesity prevention trial based on self-regulation strategy: study protocol of the Wuhan preschoolers’ healthy start project

**DOI:** 10.3389/fped.2025.1611282

**Published:** 2025-07-23

**Authors:** Wenli Dong, Yimin Wang, Ke Xu, Miyuan Wang, Wenqi Xia, Fengyan Chen, Paiziyeti Tuerxun, Yanfen Jiang, Mengna Wei, Jiameng Zhou, Jianduan Zhang

**Affiliations:** ^1^ Department of Maternal and Child Health, School of Public Health, Tongji Medical College, Huazhong University of Science and Technology, Wuhan, Hubei, China; ^2^ Key Laboratory of Environment & Health, Huazhong University of Science and Technology, Ministry of Education, Wuhan, Hubei, China

**Keywords:** childhood obesity, self-regulation, family, school, energy balance-related behavior

## Abstract

**Background:**

Childhood obesity has surged in China, with preschool years being a critical window for intervention. Despite this, evidence-based randomized trials remain scarce, and traditional prevention strategies focusing on knowledge dissemination show limited long-term efficacy. The Wuhan Preschool Healthy Start (WPHS) project addresses this gap through a kindergarten-based trial integrating self-regulation strategies with energy balance-related behavior (EBRB) interventions.

**Methods:**

This stratified randomized controlled trial enrolls children from 26 Wuhan kindergartens (13 intervention, 13 control) over 18 months. The multicomponent intervention targets diet, physical activity, sleep, sedentary behavior, and self-regulation, engaging children, families, and kindergarten environments. Control groups follow standard curricula. Primary outcome is the change in BMI z-score. Secondary outcomes assess behavioral factors (diet, sleep, activity), self-regulation skills, and other anthropometric indicators. Analyses adhere to intention-to-treat principles, using linear mixed models to evaluate intervention effects across strata while exploring potential effect modifiers including kindergarten level, child gender, and age.

**Discussion:**

This intervention hypothesizes that the integrated components of health knowledge, behavior, and self-regulation will not only support the adoption of targeted health behaviors but also ensure their long-term maintenance. This unique approach makes the WPHS project an innovative and holistic initiative to prevent childhood obesity, providing valuable insights into public health strategies for this critical population. We anticipate that incorporating self-regulation training will improve the sustainability of behavior changes, addressing a key gaps in current preschool obesity interventions.

**Trial Registration Number:**

Chinese Clinical Trial Registry ChiCTR2200058452.

## 1 Highlights

• Integrates self-regulation training to enhance the sustainability of preschool obesity prevention efforts.

• A Multicomponent intervention fosters collaboration between families and schools to promote healthy behaviors.

• This longitudinal RCT explores the role of self-regulation in shaping early health behaviors and policies.

## 2 Introduction

Childhood obesity is a pervasive global public health challenge (1, 2), with markedly rising prevalence in recent decades. In 2020, an estimated 39 million children under the age of 5 and 150 million children aged 5–19 were classified as overweight or obese (3). In China, the issue has become increasingly pressing, with the prevalence reaching 10.4% among those under six in 2020 (4), a growth rate surpassing the global average (5, 6). Childhood obesity has profound implications for both physical and psychosocial well-being. It significantly contributes to the growing burden of chronic non-communicable diseases, such as diabetes (7), cardiovascular disease (8), and gastroenterological problems (9, 10), as well as psychological issues, including depression and anxiety (11, 12), poor self-esteem, and social dysfunction (13). Moreover, childhood obesity often persists into adulthood, resulting in severe long-term outcomes such as various cancers (14, 15), premature death (16), and a substantial economic burden (17).

Preschool age represents a critical window for shaping obesity trajectory, with over 80% of children who are obese by the age of four likely to remain overweight or obese throughout adolescence (18). During this period, family (19) and kindergarten (20) environments significantly influence children's energy balance-related behaviors (EBRBs), such as physical activity, sedentary behavior, dietary habits (21), and sleep (22), which tend to become firmly established (23), laying the foundation for energy imbalance and the early onset of obesity (24). Consequently, EBERs are commonly targeted in preschool obesity prevention programs (25–28). In China, preschoolers typically spend the majority of their weekdays in kindergarten, from 8:30 am to 4:30 pm (29, 30), making these settings essential platforms for early prevention. Compared to single-component approaches, current school-based randomized controlled trials (RCTs) increasingly adopt multi-component interventions that address physical activity, diet, and health knowledge (7, 31). However, findings remain mixed and often lack long-term sustainability (32, 33). More importantly, despite short-term gains in children's health knowledge and EBRBs, these improvements often fail to translate into lasting behavior changes beyond the intervention context (34). This disconnect between immediate success and unsatisfactory long-term outcome reflects a high risk of relapse and weight regain (35, 36). This discrepancy highlights the importance of sustained motivation, which is significantly related to the level of self-regulation required over time (37). Self-regulation encompasses the control of attention, thoughts, behaviors, interpersonal interactions, and emotions (38), presenting a significant hurdle for individuals with obesity, particularly amidst daily temptations. Individuals with weak self-regulation struggle with weight loss both in the short and long term and are more likely to drop out of obesity treatment (39, 40).

Neuroimaging studies have revealed a strong association between binge eating and the brain's frontoparietal and self-regulation networks, which govern cognitive and inhibitory controls (41, 42). Clinical trials in obesity treatment have also shown that incorporating self-regulation components can effectively aid weight loss (43, 44). However, evidence regarding the effectiveness of self-regulation strategies in obesity prevention remains inconsistent. For example, a study by Robert et al. targeting healthy eating and self-regulation among toddlers aged 18–36 months reported medium to large effects on critical obesity-related factors (45). In contrast, a larger trial involving 697 children around the age of 4 that applied self-regulation strategies in a comprehensive intervention found no significant effect on obesity prevalence or related behaviors (46). These inconsistencies may stem from several methodological limitations, including small sample sizes, the absence of control groups, and brief follow-up periods, among others. Additionally, the effectiveness of an intervention may depend on how it incorporates general and food-related self-regulation components. General self-regulation typically includes emotional regulation, behavioral control, and executive cognitive processes (47, 48), while food-related self-regulation pertains to aspects of appetite control, energy intake, or the regulation of eating behaviors (49). As suggested by Lumeng et al*.,* interventions relying solely on general self-regulation may not adequately target mechanisms directly involved in childhood obesity, which could partially explain previous insignificant findings. The authors advocate for future studies to emphasize self-regulation strategies specifically tailored to food-related context (46). To date, no large-scale cluster-RCT in China has integrated both general and food-specific self-regulation training within a preschool obesity prevention framework, underscoring the innovative nature of our trial. A prevention that incorporates these two dimensions of self-regulation is expected to yield more promising outcomes.

Prior childhood obesity prevention targeting self-regulation has largely focused on isolated behaviors like diet or physical activity (50), often overlooking interconnected factors such as sleep, sedentary time, and screen use. These programs also tended to operate in single settings (e.g., home or school) (45, 51), limiting environmental reinforcement and consistency. The WPHS intervention adopts a more integrated approach, addressing multiple behaviors simultaneously - diet, exercise, sleep, sedentary habits, and screen time, while fostering home-school collaboration through caregiver education, teacher-led activities, and shared challenges. This dual-context strategy promotes consistent support across key environments, enhancing self-regulation and the potential for sustained behavior changes.

Current preschool obesity prevention efforts also face multiple broader content and design limitations.Most lack structured integration of parenting practices and underemphasize parental involvement (52, 53), missing opportunities to support family routines. Multilevel interventions often vary in delivery across settings due to resource constraints and cultural factors, reducing consistency and generalizability (54, 55). In addition, many interventions are short-term and lack sufficient follow-up periods, limiting insights into long-term outcomes (56, 57).

This study builds upon the WPHS, a kindergarten-based obesity prevention program. It aims to evaluate the effectiveness of a multi-component intervention designed to prevent childhood obesity in kindergarten settings while actively engaging families. The focus is on activities designed to enhance children's EBRBs and foster both general and food-related self-regulation, while also engaging kindergartens and families to create a supportive environment for healthy behaviors. Accordingly, the WPHS intervention targets childhood obesity through three key strategies.

First, it introduces a structured health education curriculum to convey appropriate and evidence-based messages tailored for preschoolers. This component actively involves kindergarten teachers, who are instrumental in delivering the curriculum and instilling healthy habits and lifestyles in children. Additionally, we will integrate a general and food-related self-regulation component into the curriculum, featuring specific activities and tasks to enhance children's self-regulation. This approach reinforces learned behaviors and supports the development of skills for maintaining healthy practices.

Secondly, we initiate environment-changing activities in kindergartens to foster a setting that supports healthy behaviors. This involves modifications to the physical environment (e.g., offering healthier meal options and revising activity schedules) to promote an atmosphere conducive to positive health outcomes within the kindergarten.

Thirdly, the intervention adopts a family-focused approach by providing parents with customized messages and tasks for the home, aligned with the lessons their children learn in kindergarten. The goal is to foster a supportive home environment that reinforces the healthy habits introduced through the kindergarten intervention.

Through this holistic strategy—integrating health education curricula, modifications in the kindergarten environment, and proactive involvement of families—our goal is to maximize the impact of our intervention in combating childhood obesity.

The WPHS intervention spans three semesters: a full school year (two semesters) dedicated to intensive intervention (the first and second semester of the middle year), followed maintenance phase. To assess both immediate and long-term impacts on participants’ EBRBs and adiposity, data will be collected at three critical junctures: baseline (T1, pre-intervention), post-intervention (T2, after the intensive phase), and follow-up (T3, post-program).

### 2.1 Theoretical model/ the theoretical basis

Our intervention is theoretically grounded in Social Cognitive Theory (SCT), Social Ecological Theory (SET), and the Capability, Opportunity, Motivation—Behavior (COM-B) model, each providing a complementary perspective for comprehensively addressing childhood obesity. SCT posits the reciprocal interaction among personal factors, behaviors, and the environment, highlighting the pivotal role of observational learning—children adopt new behaviors by observing role models such as parents and teachers. SET broadens this perspective by situating behavior within a broader ecological context, recognizing that the actions are shaped by interconnected systems across multiple levels, including the family, school, and community. It highlights that sustainable change requires engagement across the environmental layers. The COM-B model adds a practical lens for designing interventions by identifying three essential conditions for behavior changes: Capability (both psychological and physical), Opportunity (environmental and social), and Motivation (reflective and automatic). Change occurs when these conditions are adequately addressed.

Drawing on these frameworks, our intervention strategically targets multiple levels of influence: the individual child, their school and home environment, and the agents within those settings—teachers and parents. In the school setting, teachers serve as both educators and behavior models, shaping children's choices through structured learning and everyday interaction. At home, parents reinforce these behaviors and establish a supportive environment, promoting consistency across contexts and strengthening the intervention's impact.

### 2.2 Research hypothesis

We hypothesize that our comprehensive intervention, targeting individuals, families, and kindergartens, will catalyze positive changes in preschool children's lifestyles, leading to enhanced health knowledge, improved behaviors, and healthier weight trajectories. This outcome is expected to be achieved through the implementation of a structured health curriculum for children, covering critical areas such as diet, physical activity, sleep, and self-regulation. Home assignments corresponding to the curriculum are designed to reinforce learning outcomes and are expected to amplify the impact of the intervention. Furthermore, the overarching goal will be realized through a multifaceted approach aiming to create and fortify supportive environments both in kindergartens and at home. Key components include training teachers, providing caregivers with health-related tips, organizing themed week activities, and related initiatives.

## 3 Methods

### 3.1 Study setting

The research will take place in Jianghan and Hanyang Districts, Wuhan, the capital city of Hubei province in central China (58, 59). Wuhan, a major transportation hub with a population of ∼10 million, is one of China's largest cities. The selected districts, Jianghan and Hanyang, are two of seven central urban areas of Wuhan, with a combined population of ∼1.55 million. As of 2022, these districts house 248 kindergartens, including 67 public and 181 private institutions. As a large, representative city with a substantial population, Wuhan's childhood obesity challenges may reflect similar issues in other parts of China. Implementing interventions in cities like Wuhan can provide valuable data and insights to inform policy decisions across the country.

### 3.2 Sample size determination

The unit is randomization is the kindergarten. We calculated the sample size in Power Analysis and Sample Size (PASS) using a test for two means in a cluster-randomized design. Assuming each kindergartens contribute approximately 45 children, we determined that 11 kindergartens per arm (intervention and control) would provide 90% power (1-*β*) to detect an underlying difference of 0.28 (60) in the mean outcome (a medium standardized effect size) following the intervention. This calculation assumes an intra-class correlation coefficient (ICC) of 0.01 (61) at the two-sided significance level (*α*) of 5%. Assuming up to 20% attrition, we increased the sample to 26 kindergartens (13 intervention) (45 children/kindergarten), yielding an anticipated sample of 585 preschoolers receiving intervention.

### 3.3 Recruitment of kindergartens and participants

This research is conducted in cooperation with the Maternal and Child Health (MCH) Hospital of Jianghan and Hanyang Districts in Wuhan. A sensitization meeting was convened at the MCH Hospital to engage headmasters and doctors from selected kindergartens, where we delivered a comprehensive overview of the project objectives and discussed the planned intervention. It was critical to ensure that headmasters were thoroughly briefed on the study's purpose, and the study design was carefully crafted to align with school schedules and operations, thereby ensuring the feasibility of intervention implementation.

A total of 26 kindergartens (clusters) were selected from Jianghan and Hanyang Districts based on predefined eligibility criteria, including the willingness of the headmasters to participate, the availability of at least two junior classes, low student turnover rates, and no prior participation in similar studies.

Given the structure of the Chinese kindergarten system (which is organized into three grades: junior, middle, and senior), our intervention will enroll children in the middle grade. Within each participating kindergarten, one middle-grade class will be randomly selected, with informed consent obtained from the children's guardians before recruiting them into the project. Child participants must meet the following criteria: enrolled in the middle grade (ages 3–6 years) of participating kindergartens, provide guardian consent, reside in the study district for the full 24-month period, and have no major metabolic/cardiovascular conditions. Exclusion criteria include chronic illnesses requiring specialized care, concurrent participation in other obesity programs, or inability to complete follow-up assessments. The intervention is designed to span 18 months, commencing during the children's second year of kindergarten, and will be seamlessly integrated into daily routines in the intervention group. Following the intervention phase, there will be an additional six-month observational follow-up period.

### 3.4 Randomization and blindness

To ensure comparable representation across geographical locations, socio-economic backgrounds, and kindergarten types, cluster randomization was conducted using stratification by kindergarten level with a 1:1 allocation ratio. This approach assigns the 26 kindergartens (clusters) to either the intervention group (*n* = 13) or the control group (*n* = 13). Due to the nature of the intervention, blinding of participants and implementers is not feasible. However, to minimize potential bias, outcome assessors remain blinded to group assignments until the conclusion of data collection and initial analyses.

## 4 Intervention

The WPHS intervention program is grounded in SET, SCT, and the COM-B model, recognizing the interdependent relationship among personal characteristics, behavioral factors, and environmental influences (62). The intervention is designed to target individual children, families, and kindergartens to foster the adoption of healthy lifestyle habits and enhance children's capacities for self-regulation, aiming for sustained behavior changes and long-term effects. Table 1 outlines an overview of the intervention components and corresponding activities tailored to each target group.

**Table 1:** Main components of intervention measures.

Intervention dimensions	Intervention objectives	Accumulation of health knowledge	EBRBs practice	Self-regulation improvement
Individual	Children	Child health courses:establish health awareness and accumulate relevant knowledge in diet, physical activity, screen time, sleep and self-regulation	Family tasks: cooperate with health course to complete EBRBs tasks at home	Health-related self-regulation activities
Family	Caregivers	Health knowledge tips: develop healthy parenting concepts, gain health knowledge and skills in diet, physical activity, media use, sleep, and self-regulation	Family dietary, physical activity, and sleep guidance: researchers will instruct families on eating, physical activity, and sleep at home	
Kindergarten environment	Teachers, health care staff	Teacher training: master children's health promotion knowledge, be familiar with health courses and other teaching contents, and encourage children's healthy behaviors Guidance for health care staff: instruct food pairing in the kindergarten		

The WPHS intervention adopts a multifaceted approach to promote a balanced diet, regular physical activity, and both general and food-related self-regulation skills. A structured health education curriculum offers guidance on adopting healthy behaviors and lifestyles, complemented by behavioral challenges to encourage the long-term maintenance of these positive habits. Activities designed to bolster self-regulation empower children to make conscious, healthy choices. The intervention also emphasizes school and family engagement, incorporating teacher training sessions, dissemination of health tips to caregivers, and the organization of thematic weeks. Such collective involvement seeks to amplify the intervention's reach, emphasizing the crucial role of family support in creating a home environment that nurtures and sustains the healthy behaviors initiated at school.

**Figure 1:**
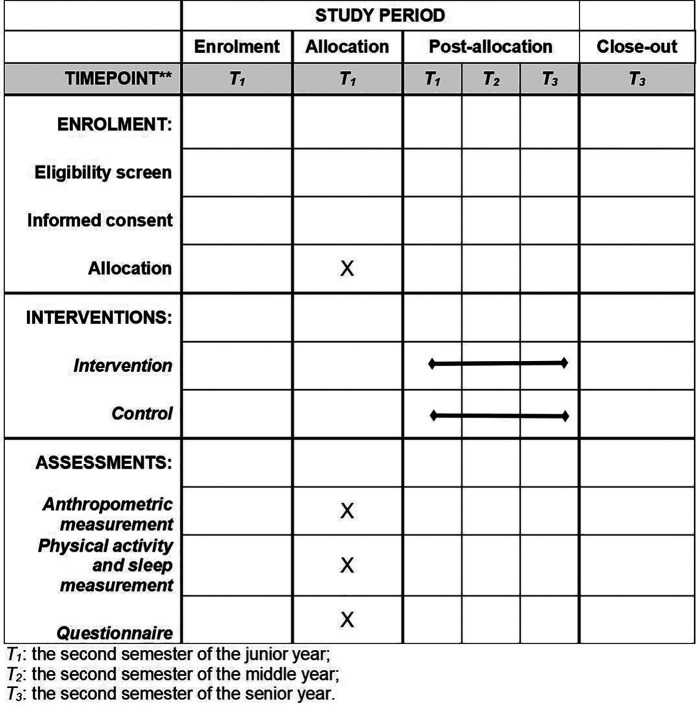
Participant timeline.

Development of the WPHS-intervention scheme underwent a rigorous three-stage process. Initially, we conducted a systematic literature review to identify core components of successful intervention. This was followed by a comprehensive cross-sectional survey conducted in 2020, involving over 4,000 preschoolers from Jianghan District, Wuhan, to gather insights into the children's lifestyle, home environment, parental feeding patterns, and self-regulation capabilities. Findings from this stage informed the tailoring of the intervention to the specific needs of our target demographic. The final stage consisted of a six-month pilot study in four kindergartens to assess the feasibility of the proposed intervention scheme in a real-world setting. Feedback from participating kindergartens and parents was instrumental in refining the study design and intervention elements. Through this iterative process, which incorporated empirical evidence, local insights, and direct feedback, the intervention was meticulously designed to ensure its applicability and impact within the local context. Figure 1 presents a participant timeline.

### 4.1 Intervention logic

**Figure 2:**
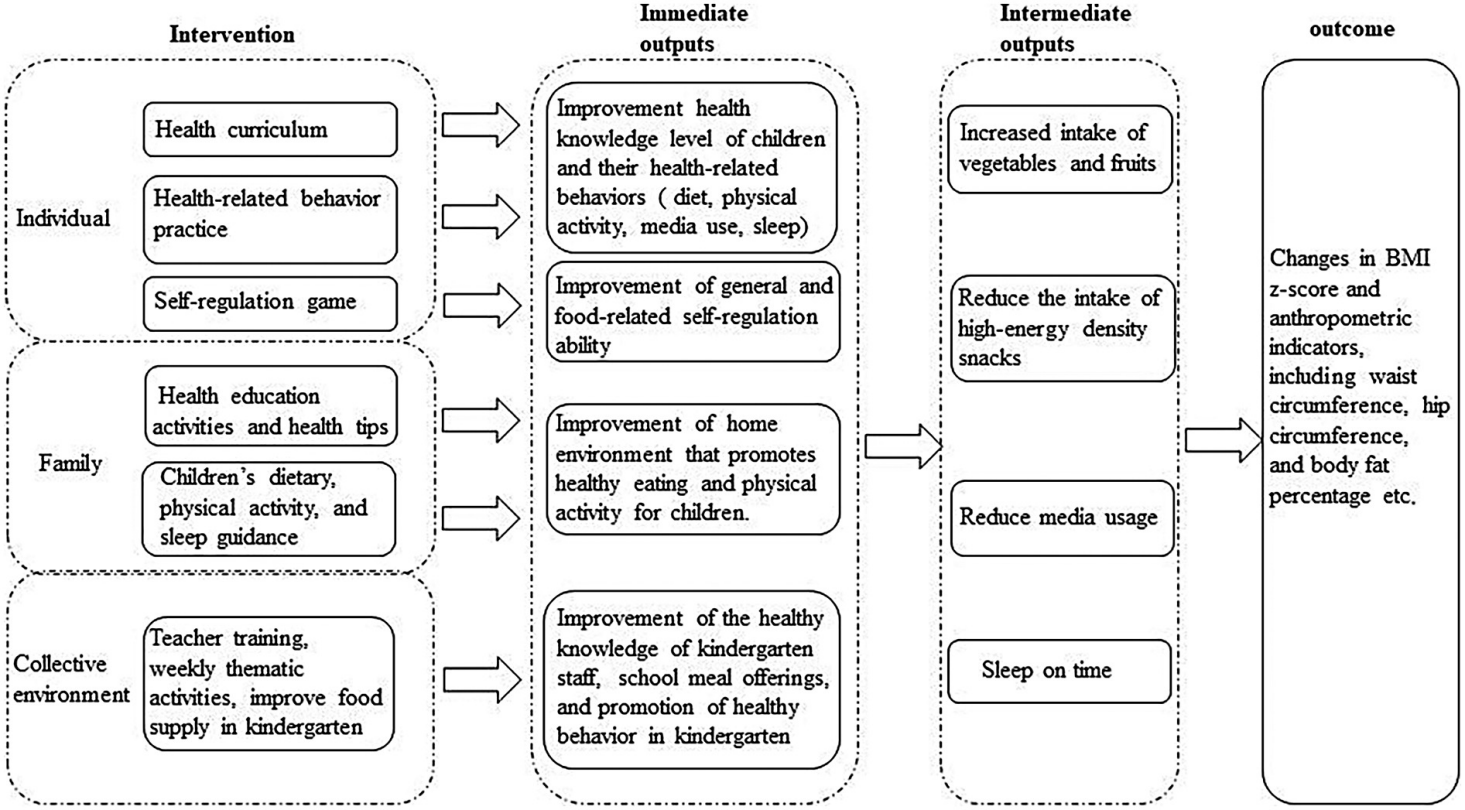
Logical model of intervention strategy.

Figure 2 illustrates the logic model underpinning our intervention strategy. By implementing this multifaceted intervention, we anticipate improvement in EBRBs and anthropometric indicators, aiming for stabilization or reduction in BMI z-scores relative to the control.

### 4.2 Individual components

#### 4.2.1 Child health curriculum in kindergarten

The health curriculum comprises 32 sessions, each lasting 15 minutes, strategically designed to enhance children's understanding and practices of healthy diet, physical activity, sleep, screen time, and self-regulation. This curriculum was developed through a rigorous multi-step approach. Initially, the research team delineated course topics based on literature review and a baseline survey, covering diet, sleep, media use, and physical activity.

A preliminary set of lesson plans and supporting PowerPoint slides (Version 1), was then crafted. Next,we held collaborative evaluations with principals, healthcare professionals, and teaching staff from two pilot kindergartens to assess feasibility and content. This process, with feedback from educational experts, led to successive refinement (Version 2 and 3). All course materials were then rigorously tested in the four pilot kindergartens and adjusted as needed to culminate in the final version (V4). The curriculum development emphasized evidence-based content that is comprehensive, feasible, and locally relevant. The courses will be delivered over three semesters, the first two for core health education content (weekly sections, intensive phase), and the final semester for reinforcement and consolidation (bi-weekly sections). Intervention class teachers will be equipped with the finalized lesson plans and trained to ensure effective delivery.

#### 4.2.2 Enhancement of self-regulation skills

This intervention incorporates activities to enhance preschoolers’ general and food-related self-regulation abilities. Research suggests the primary facets of self-regulation in this age group are inhibitory control, working memory, and emotion regulation (47, 63).

Inhibitory control is the ability to halt an ongoing action when it is considered erroneous (64). The “Happy Goat Says” activity is designed to improve children's inhibitory control in the context of food selection (65). In this game, children are introduced to three types of items: healthy food, unhealthy food, and toys. A teacher prompts specific actions with the phrase “Happy Goat says.” The children are instructed to perform the actions only when the phrase is given, practicing restraint and remaining still otherwise.

Working memory is the ability to retain and manipulate information over short periods (66). We employ an “n-back” game with pictorial stimuli (fruits or vegetables) (65). Children must determine whether the current picture matches the one shown one or two steps earlier. The difficulty adjusts based on performance, gradually increasing to challenge each child's working memory capacity.

Emotion regulation refers to managing one's initial emotional reaction (67). To enhance these skills, the intervention incorporates an emotion management segment to the health curriculum. Teachers guide children in identifying various emotions by drawing facial expressions on toy eggs and teach simple relaxation techniques to help children cope with strong emotions.

These activities, grounded in developmental science and pilot-tested in three classes, were refined based on the initial outcomes and teacher feedback to ensure they are engaging and suitable for preschoolers. The self-regulation activities are integrated into the intervention schedule over three semesters, with the first two dedicated to weekly intensive activities, and in the final semester, they are reinforced bi-weekly. Each session lasts 10–15 minutes. Teachers received training and detailed instructions to maintain fidelity to the activity protocol while allowing minor adaptations to fit classroom context.

#### 4.2.3 Promoting health-related behavior at home

The WPHS intervention extends beyond the classroom via family-centric healthy behavioral challenges, actively engaging parents in fostering a health-promoting home environment. Participating families are tasked with four distinct health challenges: (1) consuming five different fruits and vegetables daily, (2) choosing healthy snacks (e.g., milk or fruit) over high-energy-density alternatives, (3) engaging in ≥2 hours of outdoor physical activity daily, and (4) achieving 10 hours of sleep per night.

Progress is monitored through record cards that families update to track their adherence to these challenges. Trained teachers will regularly review these cards and provide feedback to both children and parents to encourage continued participation. Achievements are celebrated in the classroom with a “Reward Board” displaying badges for each child's accomplishments. Students who accumulate the most badges by the end of each term receive a special reward, further incentivizing healthy habits.

These family tasks are designed to maximize parental involvement and reinforce the health behaviors introduced in kindergarten. The challenges may be adjusted each term based on participation rates and feedback, allowing the intervention to remain responsive and effective.

### 4.3 Family-level intervention

Parents and caregivers play a crucial role in shaping healthy diet and physical activity habits in children. The WPHS-intervention actively engages families to ensure the home environment supports healthy eating and physical activity. Family involvement includes:

#### 4.3.1 Health education workshops

We will host two interactive workshops for parents (and grandparents) focusing on healthy parenting and lifestyle strategies to prevent childhood obesity. These sessions will provide practical guidance on nutrition, media use, physical activity, sleep, and strategies to foster self-regulation in children. Moreover, we have prepared a set of 37 concise health tips (delivered via handout or messaging) to reinforce essential concepts related to EBRBs and self-regulation, ensuring caregivers are well-informed and empowered to support healthy behaviors at home.

#### 4.3.2 Customizing home environment guidance

We will provide personalized feedback to each family about their child's diet, activity, and sleep patterns, along with tailored recommendations to meet national guidelines. In addition, baseline information will be collected via the Food Frequency Questionnaire (FFQ) (68), a self-developed physical activity questionnaire, and the Children's Sleep Habits Questionnaire (CSHQ). Each family will receive a summary comparing their child's habits to the Chinese Dietary Guidelines and World Health Organization (WHO) recommendations for young children. Based on this, parents will be advised on specific adjustments (e.g, reducing sugary snacks, increasing physical activity, establishing consistent bedtimes) to optimize their home environment in support of their child's health.

### 4.4 Enhancing the kindergarten environment

This component aims to improve the health knowledge of kindergarten staff, optimize the nutritional quality of school meals, and foster a health-centric atmosphere within the kindergarten. Key elements include:

#### 4.4.1 Teacher professional development

Two training sessions for teachers will increase their health literacy in the classroom and enable them to promote child health. These sessions cover topics such as nutrition, physical activity, and strategies to encourage self-regulation in the classroom. Experienced teachers from the pilot kindergartens, who helped develop the curriculum, will deliver demonstration lessons to their peers within the intervention groups. This peer-led model ensures standardized understanding of the curriculum content and teaching methodologies across educators.

#### 4.4.2 Themed weeks

Each kindergarten will implement periodic themed weeks aligned with the curriculum (e.g., “Active Play Week” or “Healthy Eating Week”). During these weeks, special activities will encourage physical activity, nutritious eating, and reduced screen time. Colorful posters aligned with the week's theme will be displayed on school bulletin boards to reinforce the health messages and generate enthusiasm among children and staff.

#### 4.4.3 Nutritional optimization of school meals

The intervention will include a review of the menus provided in the kindergartens. Researchers will analyze the nutrients in school meals and provide recommendations to improve their nutritional balance. The intervention aims to align meals with the Dietary Guidelines for Chinese Preschool-aged Children (2022) issued by the Chinese Nutrition Society (69).

### 4.5 Intervention outcomes

Evaluation of the WPHS intervention will focus on a comprehensive set of primary and secondary outcomes. The primary outcome is BMI z-score. Secondary outcomes include: (1) dietary behaviors (e.g., weekly intake of high-energy-density foods and sugar-sweetened beverages, and daily servings of fruits and vegetables), (2) sleep-related outcomes (e.g., average nightly sleep duration and sleep quality), (3) physical activity levels (e.g., time in moderate-to-vigorous physical activity and sedentary time), and (4) general and food-related self-regulation indicators (e.g., performance on the day-night task, less-is-more task, Corsi block-tapping task, digit span task, and scores on the Childhood Executive Functioning Inventory (CHEXI), and Children's Behaviour Questionnaire (CBQ) (5) other anthropometric indicators, including waist circumference, hip circumference, and body fat percentage (70).

## 5 Data collection

Table 2 provides an assessment of metrics, methodologies, and data collection schedules used in the study. Trained research personnel will follow standardized protocols and utilize validated tools to ensure data accuracy and consistency across all time points. Assessments were conducted at three key intervals: T1 (baseline) in the second semester of the junior year; T2 (post-intervention) in the second semester of the mid-year; and T3 (follow-up) in the second semester of the senior year.

**Table 2:** Overview of measurement indicators, instruments, and time point of assessment.

Data collection	Indicators	Assessment instruments	Time points of assessment
Measurement in kindergartens	• Height and weight	• Kangwa WS-RT-2U	• T1,T2,T3
	• Waist and hip circumference	• Measuring tape	• T1,T2,T3
	• Body fat percentage	• Multifrequency bioelectric impedance body composition analyzer Inbody 230	• T1,T2,T3
	• Skinfold thickness^a^	• Harpenden Skinfold calipers	• T1,T2,T3
	• Self-regulation level	• Self-regulation related tests	• T1, T2, T3
Actigraph GT3X+	• Physical activity and sleep^a^	• Actigraph GT3X+	• T1, T3
Questionnaire	• Basic information		• T1
	• Dietary behavior	• CEBQ (46), FFQ (48)	• T1,T2,T3
	• Sleep quality in children	• CSHQ (50)	• T1,T2,T3
	• General self-regulation level	• CBQ (51)	• T1,T2,T3
	• Food-related self-regulation level	• CEBQ (46)	• T1,T2,T3
	• Psychological behavior of children	• CBQ (51)	• T1,T2,T3

T1, the second semester of the junior year; T2, the second semester of the middle year; T3, the second semester of the senior year; CEBQ, Children's Eating Behavior Questionnaire; FFQ, food frequency questionnaire; CSHQ, Children's Sleep Habit Questionnaire; CBQ, Children's Behavior Questionnaire.

^a^ These indicators will be measured for some children but not all.

### 5.1 Anthropometric measurement

Weight and height will be measured using calibrated scales and stadiometers [e.g., Kangwa (WS-RT-2U), accurate to 0.01 kg for weight and 0.1 cm for height]. To ensure reliability, each measurement will be taken twice. If discrepancies greater than 0.5 cm in height or 0.1 kg in weight are observed, a third measurement will be taken until the differences between any two consecutive readings fall below these thresholds. The average of the valid measurements will be used for subsequent analysis. Body composition will be estimated through Bioelectrical Impedance Analysis (InBody 230 device), which provides resistance readings used to calculate body fat percentage, fat-free mass, and fat mass.

Table 3 outlines the four tests of self-regulation used in the study, and their scoring. All fieldworkers will undergo comprehensive training to ensure standardized procedures across data collection.

**Table 3:** The name and description of self-regulation-related tests.

Self-regulation	Name	Description
Inhibitory control	• Stroop test-Day and night (52)	Instruct children to respond in the opposite manner when answering questions, and present a series of cards with the sun or moon. In the presentation of a sun card, instruct children to respond with “night”, and when a card with stars and the moon is displayed, prompt children to answer “day”.
	• Less is more (53)	For food-related tasks, researchers will display various food toys and ask children to choose their preferred item for the experiment. The child will be prompted to indicate a bowl of food toys (containing 2 or 5 items), and the researcher will collect the chosen toys. Conversely, the child will receive the food toys they did not point to during the selection process.
	• Modified Stroop Task	In each trial, children were presented with various numbers of cherries and watermelons at the same time, and they were asked to indicate which set had more than the other, irrespective of fruit size. There were 64 trials per task variant, of which 50% congruent trials (where the larger number was represented by larger-sized stimuli, e.g., two cherries vs. five watermelons) and 50% incongruent trials (e.g., five cherries vs. two watermelons). The congruent trials and incongruent trials were randomly intermixed.
Working memory	• Corsi Block tapping task (54)	The researchers tap the blocks in a specific order, and the children are instructed to tap them in the same order and in reverse order. The difficulty of the test is progressively increased by sequentially adding more tapping blocks.
	• Digit span test (55)	This test involves a number table ranging from 2 digits to 13 digits. The researchers read a list of numbers, and the children are required to recite them both in order and in reverse order.
Executive function	• Childhood Executive Function Inventory	It consists of 24-items and comprises four scales: Inhibit, Shift, Working Memory, and Plan/Organize. Participants rate items on a five-point scale (i.e., Definitely not true, Not true, Partially true, True, Definitely true), with higher scores indicating lower executive function.

### 5.2 Physical activity and sleep measurement

A subset of participants will be randomly selected to wear the Actigraph GT3X + accelerometer on the non-dominant wrist for seven consecutive days to objectively measure physical activity and sleep patterns. If any child fails to wear the device for 7 days, or if device issues arise, an additional week of monitoring will be scheduled for that child. The accelerometer data will be used to derive measures such as average daily activity counts, time spent in moderate-to-vigorous activity, sedentary time, and sleep duration (using validated cut-points and algorithms).

### 5.3 Questionnaires

A structured questionnaire will capture demographic details and assess behaviors not captured by devices. This includes dietary intake [*via* the FFQ (71), CEBQ (72), sleep habits *via* the CSHQ (73), CHEXI (74), and the CBQ (75)]. These instruments are established measures with appropriate validity for our population.

## 6 Quality control

A rigorous process evaluation will be conducted throughout the intervention to ensure it is delivered with high fidelity and reaches the target population effectively. We focus on three key elements: Recruitment/Reach, Dose Delivered (Completeness), and Fidelity.

For Recruitment/Reach, we will document recruitment and participation rates by tracking the number and characteristics of kindergartens and families that consent, alongside those who withdraw or are lost to follow-up. This will help identify any biases in reach and ensure the intervention is engaging the intended demographic. For Dose Delivered (Completeness), we will record the frequency and duration of all intervention activities (e.g., number of curriculum sessions taught, number of family challenge cards distributed and completed). We will also track the extent to which each component is implemented (for instance, tallying how many of the 32 curriculum sessions were delivered in each class, and how many health tips and posters were distributed as planned). Specifically, we will document the distribution of intervention materials, including 4 family task cards, 37 health tips, and 11 posters, to confirm they were provided to parents/caregivers and displayed in kindergartens as intended. For fidelity, we will verify that the intervention is executed according to the protocol. This includes tracking that children's behavioral challenge records are maintained, that classroom activities follow the lesson plan, and family engagement components occur on schedule. Trained evaluators will use checklists during random, unannounced visits to observe a sample of intervention sessions and ensure adherence to content and methods.

The process evaluation employs brief questionnaires with Likert-scale and open-ended items for teachers and parents to provide feedback on each component. This approach is designed to be time-efficient and not overly burdensome, while still providing comprehensive and quantifiable data. Table 4 presents a detailed overview of these process evaluation tools.

**Table 4:** The process evaluation tools used in the WPHS-intervention.

Process evaluation tools	Process evaluation elements	Information/data collection
Recruitment log	Recruitment procedures	• Weekly monitoring of recruited kindergartens. • Tracking the number of kindergartens and children approached, those participated in baseline, entered the WPHS-intervention, and in the follow-up evaluation. • Documenting characteristics of kindergartens/children dropping out.
Teacher's Lesson Assessment Questionnaire	Dose delivered (completeness)	• Recording date, duration, quantity, and type of classroom sessions.
	Fidelity	• Noting any deviations or modifications made to original lesson plan. • Recording the start and end times of schedule activities. • Observing any unexpected changes or factors influencing adherence to the plan.
	Dose received (exposure and satisfaction)	• Teachers’ feedback on the comprehensibility, readability, relevance, credibility, attractiveness of the teaching materials. • Teachers’ feedback on children's behavior change with the WPHS project as implemented in the classroom. • Midpoint feedback from teachers regarding suggestions for improving the WPHS program and materials. • Teachers’ feedback on the barriers and facilitators for implementing each part of the intervention.
Attendance log	Dose received (exposure)	• Daily records of children's attendance in the kindergarten. • Identification of days when the WPHS-intervention activities are conducted. • Recording any factors affecting attendance.
Teachers’ post-intervention questionnaire	Dose received (exposure and satisfaction)	• Teachers’ reflections on the barriers and facilitators during the implementation of the WPHS project. • Feedback on the feasibility, attractiveness of the WPHS project. • Subjective feedback regarding observed behavioral changes in children's. • Subjective feedback on parents’, teachers’ and personnel's satisfaction with the WPHS project.
Teachers’ mid-intervention questionnaire	Dose received (exposure and satisfaction)	• Teachers’ reflections on the barriers and facilitators during the implementation of the WPHS project. • Feedback on the feasibility, attractiveness of the WPHS project. • Subjective feedback regarding observed behavioral changes in children. • Subjective feedback on parents’, teachers’ and personnel's satisfaction with the WPHS project.
Parent's post-intervention questionnaire	Dose received (exposure and satisfaction)	• Parents’ confirmation of receiving and/or reading each part of the material (tips, posters, family task cards). • Parents’ satisfaction with the WPHS project. • Parents feedback regarding the implementation of activities included in the material (family task cards) with their children at home. • Subjective feedback on observed behavioral changes in their child with the WPHS project. • Feedback was collected on the comprehensibility, readability, relevance, credibility, and attractiveness of the material (tips, posters, family task cards).
Parent's mid-intervention questionnaire	Dose received (exposure and satisfaction)	• Parents’ confirmation of receiving and/or reading each part of the material (tips, posters, family task cards). • Parents’ satisfaction with the WPHS project. • Parents feedback regarding the implementation of activities included in the material (family task cards) with their children at home. • Subjective feedback on observed behavioral changes in their child with the WPHS project. • Feedback on the comprehensibility, readability, relevance, credibility, and attractiveness of the material (tips, posters, family task cards).
Audit questionnaire	Physical and social environment, and the context of WHSP intervention	• Information on school food and physical activity environment (i.e., infrastructure/facilities/equipment, curriculum and personnel/health-related programmes in progress, which were related to the healthy lifestyle behaviours).
Researchers’ evaluation form	Dose delivered (completeness)	• We recorded the number and duration of training sessions
	Fidelity	• Venue of training. • Researchers who performed each training. • Records of the content of teacher training.
	Dose received (exposure and satisfaction)	• Numbers of teachers invited and attended. • Subjective assessment of teachers’ satisfaction with each training by the researchers who implemented the training.

## 7 Statistical analysis plan

The statistical analyses will adhere to the intention-to-treat principle. Participants’ characteristics will be described by study arm. Continuous variables will be presented as mean (standard deviation) or median (interquartile range), depending on distribution, and compared between groups using Student's t-tests for normally distributed data or the Wilcoxon rank-sum test for non-normal variables. Categorical variables will be reported as frequency (%) and compared using the Chi-square test. To address missing data, multiple imputation by chained equations will be used to impute covariates.

The primary analysis will evaluate the impact of the intervention on the primary outcome (e.g., changes in BMI z-score) using a linear mixed-effects regression model. Because BMI z-scores are measured repeatedly for each child at multiple time points (T1-T3), the data have a hierarchical structure, with repeated measures nested within children and children nested within kindergartens. Linear mixed-effects models are therefore employed to account for within-subject correlations over time and clustering by kindergarten, by including random intercepts for both children and kindergartens. For dichotomous outcomes, logistic mixed-effects models will be employed. These models will incorporate a study group (intervention vs. control), and relevant covariates (kindergarten level, child age and sex, and maternal sociodemographic characteristics). The models will also account for the clustered design, and random intercepts for kindergarten and children will be included. Furthermore, the potential mediating role of self-regulation on the association between the intervention and weight status will be explored.

Stratified analysis will examine whether the intervention effect varies across different subgroups. Potential effect modifiers include kindergarten level, child gender, and child age.

All hypothesis tests will use a two-sided *α* = 0.05.

## 8 Discussion

The Wuhan Healthy Start Project is a cluster-randomized trial conducted in two central districts of Wuhan City, designed to evaluate the effectiveness of the WPHS intervention among children aged 3–6 years. The intervention adopts a multifaceted approach, targeting individual children, their families, and kindergarten environments to foster healthy behaviors. A pilot testing was conducted prior to the main trial to refine intervention measures, ensuring their feasibility, cultural relevance, and suitability for the large-scale trial. The WPHS intervention advocates a spectrum of healthy habits, including nutritious eating, regular physical activity, and reduced sedentary behaviors, with a core emphasis on enhancing self-regulation skills. This focus is anticipated to support the sustained adoption of healthy behaviors beyond the intervention period. Spanning eighteen months, the program aims to foster durable behavioral changes and facilitate healthy weight progression.

Unlike prior childhood obesity interventions (76, 77) that often relied on extrinsic motivators (e.g., rewards for compliance) or isolated skill-building without environmental reinforcement (32, 33), the WPHS integrates general self-regulation strategies (e.g., goal-setting, impulse control) with context-specific applications (e.g., mindful eating, screen time budgeting). This approach fosters intrinsic motivation and adaptive capacity—critical for long-term habit formation. Crucially, the intervention targets multiple interrelated behaviors, diet, activity, sleep, and sedentary time, acknowledging their interconnected nature and enabling children to apply self-regulation across diverse daily situations (50, 51). The dual-context delivery model, involving both home and school environments, reinforces these skills in complementary settings, addressing the “contextual fragility” seen in prior single-setting interventions. We hypothesize that improvements in self-regulation will mediate the intervention's long-term effects by promoting autonomy and resilience against obesogenic triggers. This mechanism will be formally examined in planned mediation analyses. This approach is grounded in developmental theory, which posits that self-regulation, when nurtured within supportive ecosystems, becomes a transferable lifelong asset, overcoming limitations of short-term, behavior-specific paradigms. One unique aspect of this WPHS intervention is its integration of specialized training to strengthen children's self-regulation abilities, recognizing that sustained behavior change depends on skills that are transferable beyond the intervention setting. The large sample size of this study enhances the statistical power and external validity of its findings. Moreover, close collaboration with the district MCH Hospital has facilitated effective implementation, enhancing engagement from both kindergartens and caregivers while helping to minimize participant attrition. Despite its strengths, the study faces potential challenges, including participant withdrawal due to school transfers or kindergarten closures. To mitigate this, researchers will maintain detailed records of withdrawn children and, where feasible, conduct follow-up assessments to capture outcome data. Another consideration is the variations in teaching styles and implementation fidelity across classrooms, which may impact intervention effectiveness. To address these effects, we have established a stringent protocol for monitoring intervention delivery, and will continuously evaluate the process indicators to minimize bias and ensure consistency (see Quality Control Section). In conclusion, the WPHS trial represents an innovative framework by integrating a traditional multi-component obesity intervention with targeted self-regulation skill development in early childhood. The dual-focus approach is expected to yield more sustainable behavior change and improved weight-related outcomes compared to conventional models. If successful, the findings from this trial could have significant implications for the design of future childhood obesity prevention programs and public health policies, both within China and similar preschool settings globally—by highlighting the critical role of self-regulation as a foundational element in long-term obesity prevention.

## References

[1] NCD Risk Factor Collaboration (NCD-RisC). Worldwide trends in body-mass index, underweight, overweight, and obesity from 1975 to 2016: a pooled analysis of 2416 population-based measurement studies in 128·9 million children, adolescents, and adults. Lancet. (2017) 390:2627–42. 10.1016/S0140-6736(17)32129-3 29029897 PMC5735219

[2] Jia P Xue H Zhang J Wang Y. Time trend and demographic and geographic disparities in childhood obesity prevalence in China-evidence from twenty years of longitudinal data. Int J Environ Res Public Health. (2017) 14(4):369. 10.3390/ijerph14040369 28362361 PMC5409570

[3] Ng M Fleming T Robinson M Thomson B Graetz N Margono C Global, regional, and national prevalence of overweight and obesity in children and adults during 1980-2013: a systematic analysis for the global burden of disease study 2013. Lancet. (2014) 384:766–81. 10.1016/S0140-6736(14)60460-8 24880830 PMC4624264

[4] Chinese National Health and Family Planning Commission. N.H.C.J.B.P.s.M.P. House, Report on nutrition and chronic disease status of Chinese residents. (2020).

[5] Pan XF Wang L Pan A. Epidemiology and determinants of obesity in China. Lancet Diabetes Endocrinol. (2021) 9:373–92. 10.1016/S2213-8587(21)00045-0 34022156

[6] Reilly JJ. Health effects of overweight and obesity in 195 countries. N Engl J Med. (2017) 377:1496. 10.1056/NEJMc1710026 29020585

[7] Bhargava SK Sachdev HS Fall CHD Osmond C Lakshmy R Barker DJP Relation of serial changes in childhood body-mass index to impaired glucose tolerance in young adulthood. N Engl J Med. (2004) 350:865–75. 10.1056/NEJMoa035698 14985484 PMC3408694

[8] Cardiovascular risk factors and excess adiposity among overweight children and adolescents: the bogalusa heart study. J Pediatr. (2007) 150:12–17.e2. 10.1016/j.jpeds.2006.08.042 17188605

[9] Faienza MF Wang DQ Frühbeck G Garruti G Portincasa P. The dangerous link between childhood and adulthood predictors of obesity and metabolic syndrome. Intern Emerg Med. (2016) 11:175–82. 10.1007/s11739-015-1382-6 26758061

[10] Nathan BM Moran A. Metabolic complications of obesity in childhood and adolescence: more than just diabetes. Curr Opin Endocrinol Diabetes Obes. (2008) 15:21–9. 10.1097/MED.0b013e3282f43d19 18185059

[11] Childhood obesity: a review of increased risk for physical and psychological comorbidities. Clin Ther. (2013) 35:A18–32. 10.1016/j.clinthera.2012.12.014 23328273 PMC3645868

[12] Geel MV Vedder P Tanilon J. Are overweight and obese youths more often bullied by their peers? A meta-analysis on the relation between weight status and bullying. Int J Obes (Lond). (2014) 38(10):1263–7. 10.1038/ijo.2014.117 25002148

[13] Daniels SR Arnett DK Eckel RH Gidding SS Hayman LL Kumanyika S Overweight in children and adolescents: pathophysiology, consequences, prevention, and treatment. Circulation. (2005) 111(15):1999–2012. 10.1161/01.CIR.0000161369.71722.10 15837955

[14] Bertrand KA Giovannucci E Zhang SM Laden F Rosner B Birmann BM. A prospective analysis of body size during childhood, adolescence, and adulthood and risk of non-hodgkin lymphoma. Cancer Prev Res (Phila). (2013) 6:864–73. 10.1158/1940-6207.CAPR-13-0132 23803416 PMC3761937

[15] Nogueira L Stolzenberg-Solomon R Gamborg M Sørensen TIA Baker JL. Childhood body mass index and risk of adult pancreatic cancer. Curr Dev Nutr. (2017) 1(10):e001362. 10.3945/cdn.117.001362 29388617 PMC5788457

[16] Halade GV Kain V. Obesity and cardiometabolic defects in heart failure pathology. Compr Physiol. (2017) 7:1463–77. 10.1002/j.2040-4603.2017.tb00788.x 28915332 PMC5903473

[17] Withrow D Alter DA. The economic burden of obesity worldwide: a systematic review of the direct costs of obesity. Obes Rev. (2011) 12:131–41. 10.1111/j.1467-789X.2009.00712.x 20122135

[18] Geserick M Vogel M Gausche R Lipek T Spielau U Keller E Acceleration of BMI in early childhood and risk of sustained obesity. N Engl J Med. (2018) 379:1303–12. 10.1056/NEJMoa1803527 30281992

[19] Golan M Crow S. Parents are key players in the prevention and treatment of weight-related problems. Nutr Rev. (2004) 62:39–50. 10.1111/j.1753-4887.2004.tb00005.x 14995056

[20] Goldfield GS Alysha H Kimberly G Adamo KB. Physical activity promotion in the preschool years: a critical period to intervene. Int J Environ Res Public Health. (2012) 9(4):1326–42. 10.3390/ijerph9041326 22690196 PMC3366614

[21] Bandelli LN Gray HL Paul RC Contento IR Koch PA. Associations among measures of energy balance related behaviors and psychosocial determinants in urban upper elementary school children. Appetite. (2017) 108:171–82. 10.1016/j.appet.2016.09.027 27677854

[22] Must A Parisi SM. Sedentary behavior and sleep: paradoxical effects in association with childhood obesity. Int J Obes. (2009) 33:S82–6. 10.1038/ijo.2009.23 PMC3586418 19363515

[23] Birch LL Anzman SL. Learning to eat in an obesogenic environment: a developmental systems perspective on childhood obesity. Child Dev Perspect. (2010) 4:138–43. 10.1111/j.1750-8606.2010.00132.x

[24] Kremers SPJ Visscher TLS Seidell JC Vanmechelen W Brug J. Cognitive determinants of energy balance-related behaviours: measurement issues. Sports Med. (2005) 35(11):923–33. 10.2165/00007256-200535110-00001 16271007

[25] Colquitt JL Loveman E O'Malley C Azevedo LB Rees K. Diet, physical activity, and behavioural interventions for the treatment of overweight or obesity in preschool children up to the age of 6 years. Cochrane Database Syst Rev. (2016) 3:CD012105. 10.1002/14651858.CD012105 26961576 PMC6669248

[26] Liu Z Xu HM Wen LM Peng YZ Wang HJ. A systematic review and meta-analysis of the overall effects of school-based obesity prevention interventions and effect differences by intervention components. Int J Behav Nutr Phys Act. (2019) 16:95. 10.1186/s12966-019-0848-8 31665040 PMC6819386

[27] Wang Y Cai L Wu Y Wilson RF Weston C Fawole O What childhood obesity prevention programmes work? A systematic review and meta-analysis. Obes Rev. (2015) 16(7):547–65. 10.1111/obr.12277 25893796 PMC4561621

[28] Kim J Kim G Park J Wang Y Lim H. Effectiveness of teacher-led nutritional lessons in altering dietary habits and nutritional Status in preschool children: adoption of a NASA mission X-based program. Nutrients. (2019) 11:1590. 10.3390/nu11071590 31337047 PMC6682966

[29] Ickes MJ McMullen J Haider T Sharma M. Global school-based childhood obesity interventions: a review. Int J Environ Res Public Health. (2014) 11:8940–61. 10.3390/ijerph110908940 25170684 PMC4198999

[30] Bleich SN Vercammen KA Zatz LY Frelier JM Ebbeling CB Peeters A. Interventions to prevent global childhood overweight and obesity: a systematic review. Lancet Diabetes Endocrinol. (2018) 6:332–46. 10.1016/S2213-8587(17)30358-3 29066096

[31] Psaltopoulou T Tzanninis S Ntanasis-Stathopoulos I Panotopoulos G Kostopoulou M Tzanninis IG Prevention and treatment of childhood and adolescent obesity: a systematic review of meta-analyses. World J Pediatr. (2019) 15:350–81. 10.1007/s12519-019-00266-y 31313240

[32] Lyn R Heath E Dubhashi J. Global implementation of obesity prevention policies: a review of progress, politics, and the path forward. Curr Obes Rep. (2019) 8:504–16. 10.1007/s13679-019-00358-w 31673982

[33] Chen Y Henson S Jackson AB Richards JS. Obesity intervention in persons with spinal cord injury. Spinal Cord. (2006) 44:82–91. 10.1038/sj.sc.3101818 16103891

[34] Weygandt M Mai K Dommes E Ritter K Leupelt V Spranger J Impulse control in the dorsolateral prefrontal cortex counteracts post-diet weight regain in obesity. Neuroimage. (2015) 109:318–27. 10.1016/j.neuroimage.2014.12.073 25576647

[35] Bray GA Kim KK Wilding JPH. Obesity: a chronic relapsing progressive disease process. A position statement of the world obesity federation. Obes Rev. (2017) 18:715–23. 10.1111/obr.12551 28489290

[36] Reinehr T Widhalm K l'Allemand D Wiegand S Wabitsch M Holl RW. Two-year follow-up in 21,784 overweight children and adolescents with lifestyle intervention. Obesity (Silver Spring). (2009) 17:1196–9. 10.1038/oby.2009.17 19584877

[37] Kwasnicka D Dombrowski SU White M Sniehotta F. Theoretical explanations for maintenance of behaviour change: a systematic review of behaviour theories. Health Psychol Rev. (2016) 10:277–96. 10.1080/17437199.2016.1151372 26854092 PMC4975085

[38] Hofmann W Schmeichel BJ Baddeley AD. Executive functions and self-regulation. Trends Cogn Sci. (2012) 16:174–80. 10.1016/j.tics.2012.01.006 22336729

[39] Nederkoorn C Jansen E Mulkens S Jansen A. Impulsivity predicts treatment outcome in obese children. Behav Res Ther. (2007) 45:1071–5. 10.1016/j.brat.2006.05.009 16828053

[40] Anderson SE Whitaker RC. Association of self-regulation with obesity in boys vs girls in a US national sample. JAMA Pediatr. (2018) 172:842–50. 10.1001/jamapediatrics.2018.1413 30014141 PMC6143067

[41] Park BY Lee MJ Kim M Kim SH Park H. Structural and functional brain connectivity changes between people with abdominal and non-abdominal obesity and their association with behaviors of eating disorders. Front Neurosci. (2018) 12:741. 10.3389/fnins.2018.00741 30364290 PMC6193119

[42] Park BY Seo J Park H. Functional brain networks associated with eating behaviors in obesity. Sci Rep. (2016) 6:23891. 10.1038/srep23891 27030024 PMC4814917

[43] Frie K Hartmann-Boyce J Jebb SA Aveyard P. Effectiveness of a self-regulation intervention for weight loss: a randomized controlled trial. Br J Health Psychol. (2020) 25:652–76. 10.1111/bjhp.12436 32489005

[44] Hayes JF Eichen DM Barch DM Wilfley DE. Executive function in childhood obesity: promising intervention strategies to optimize treatment outcomes. Appetite. (2018) 124:10–23. 10.1016/j.appet.2017.05.040 28554851 PMC5702584

[45] Nix RL Francis LA Feinberg ME Gill S Jones DE Hostetler ML Improving Toddlers’ healthy eating habits and self-regulation: a randomized controlled trial. Pediatrics. (2021) 147(1):e20193326. 10.1542/peds.2019-3326 33372118 PMC7780956

[46] Lumeng JC Miller AL Horodynski MA Brophy-Herb HE Contreras D Lee H Improving self-regulation for obesity prevention in head start: a randomized controlled trial. Pediatrics. (2017) 139:e20162047. 10.1542/peds.2016-2047 28557722

[47] Nigg JT. Annual research review: on the relations among self-regulation, self-control, executive functioning, effortful control, cognitive control, impulsivity, risk-taking, and inhibition for developmental psychopathology. J Child Psychol Psychiatry. (2017) 58:361–83. 10.1111/jcpp.12675 28035675 PMC5367959

[48] Anderson SE Keim SA. Parent-child interaction, self-regulation, and obesity prevention in early childhood. Curr Obes Rep. (2016) 5:192–200. 10.1007/s13679-016-0208-9 27037572 PMC4856567

[49] Russell CG Russell A. “Food” and “non-food” self-regulation in childhood: a review and reciprocal analysis. Int J Behav Nutr Phys Act. (2020) 17:33. 10.1186/s12966-020-00928-5 32151265 PMC7063723

[50] Vermeiren E Naets T Van Eyck A Vervoort L Ysebaert M Baeck N Improving treatment outcome in children with obesity by an online self-control training: a randomized controlled trial. Front Pediatr. (2021) 9:794256. 10.3389/fped.2021.794256 35004547 PMC8733681

[51] Naets T Vervoort L Ysebaert M Van Eyck A Verhulst S Bruyndonckx L WELCOME: improving WEight controL and CO-morbidities in children with obesity via executive function training: study protocol for a randomized controlled trial. BMC Public Health. (2018) 18:1075. 10.1186/s12889-018-5950-3 30157826 PMC6116429

[52] Birch LL Ventura AK. Preventing childhood obesity: what works? Int J Obes. (2005) 33(Suppl 1):S74–81. 10.1038/ijo.2009.22 19363514

[53] Crawford PB Gosliner W Anderson C Strode P Becerra-Jones Y Samuels S Counseling latina mothers of preschool children about weight issues: suggestions for a new framework. J Am Diet Assoc. (2004) 104:387–94. 10.1016/j.jada.2003.12.018 14993861

[54] Gómez SF Casas Esteve R Subirana I Serra-Majem L Fletas Torrent M Homs C Effect of a community-based childhood obesity intervention program on changes in anthropometric variables, incidence of obesity, and lifestyle choices in Spanish children aged 8 to 10 years. Eur J Pediatr. (2018) 177:1531–9. 10.1007/s00431-018-3207-x 30027297

[55] Gregory JW. Prevention of obesity and metabolic syndrome in children. Front Endocrinol (Lausanne). (2019) 10:669. 10.3389/fendo.2019.00669 31632348 PMC6779866

[56] Narzisi K Simons J. Interventions that prevent or reduce obesity in children from birth to five years of age: a systematic review. J Child Health Care. (2021) 25:320–34. 10.1177/1367493520917863 32295414 PMC8076837

[57] Nordlund S McPhee PG Gabarin R Deacon C Mbuagbaw L Morrison KM. Effect of obesity treatment interventions in preschool children aged 2–6 years: a systematic review and meta-analysis. BMJ Open. (2022) 12:e053523. 10.1136/bmjopen-2021-053523 35383062 PMC8984001

[58] Kwwa B Jie GC Xxs D Jiang HE Hua WB Xlw A Fangcang shelter hospitals are a one health approach for responding to the COVID-19 outbreak in Wuhan, China - ScienceDirect. One Health. (2020) 10:100167. 10.1016/j.onehlt.2020.100167 33117879 PMC7582216

[59] Luo W Yao J Mitchell R Zhang X. Spatiotemporal access to emergency medical services in Wuhan, China: accounting for scene and transport time intervals. Int J Health Geogr. (2020) 19(1):52. 10.1186/s12942-020-00249-7 33243272 PMC7689650

[60] Dukhi N Sartorius B Taylor M. A behavioural change intervention study for the prevention of childhood obesity in South Africa: protocol for a randomized controlled trial. BMC Public Health. (2020) 20:179. 10.1186/s12889-020-8272-1 32019551 PMC7001200

[61] Deckert A Anders S de Allegri M Nguyen HT Souares A McMahon S Effectiveness and cost-effectiveness of four different strategies for SARS-CoV-2 surveillance in the general population (CoV-surv study): a structured summary of a study protocol for a cluster-randomised, two-factorial controlled trial. Trials. (2021) 22:39. 10.1186/s13063-020-04982-z 33419461 PMC7791150

[62] Anton S Das SK McLaren C Roberts SB. Application of social cognitive theory in weight management: time for a biological component? Obesity (Silver Spring). (2021) 29:1982–6. 10.1002/oby.23257 34705335 PMC8612961

[63] Blair C. Developmental science and executive function. Curr Dir Psychol Sci. (2016) 25:3–7. 10.1177/0963721415622634 26985139 PMC4789148

[64] Aron RA. The neural basis of inhibition in cognitive control. Neuroscientist. (2007) 13:214–28. 10.1177/1073858407299288 17519365

[65] Jiang Q He D Guan W He X. “Happy goat says”: the effect of a food selection inhibitory control training game of children’s response inhibition on eating behavior. Appetite. (2016) 107:86–92. 10.1016/j.appet.2016.07.030 27457969

[66] Sreenivasan KK Curtis CE D'Esposito M. Revisiting the role of persistent neural activity during working memory. Trends Cogn Sci. (2014) 18:82–9. 10.1016/j.tics.2013.12.001 24439529 PMC3964018

[67] Archi SE Cortese S Ballon N Réveillère C Brunault P. Negative affectivity and emotion dysregulation as mediators between ADHD and disordered eating: a systematic review. Nutrients. (2020) 12(11):3292. 10.3390/nu12113292 33121125 PMC7693832

[68] Saravia L Miguel-Berges ML Iglesia I Nascimento-Ferreira MV Perdomo G Bove I Relative validity of FFQ to assess food items, energy, macronutrient and micronutrient intake in children and adolescents: a systematic review with meta-analysis. Br J Nutr. (2021) 125(7):792–818. 10.1017/S0007114520003220 32807247

[69] C.N. Society, Dietary Guidelines for Chinese preschool-aged Children (2022), (2022).

[70] Bloem M. The 2006 WHO child growth standards. Br Med J. (2007) 334:705–6. 10.1136/bmj.39155.658843.BE 17413142 PMC1847861

[71] Xu K Zhang Y Dong W Tuerxun P Li C Chang R The mediation role of health behaviors in the association between self-regulation and weight Status among preschool children: a sex-specific analysis. Nutrients. (2022) 14(9):1692. 10.3390/nu14091692 35565663 PMC9104780

[72] Wardle J Guthrie CA Sanderson S Rapoport L. Development of the children’s eating behaviour questionnaire. J Child Psychol Psychiatry. (2001) 42:963–70. 10.1111/1469-7610.00792 11693591

[73] Zaidman-Zait A Zwaigenbaum L Duku E Bennett T Szatmari P Mirenda P Factor analysis of the children’s sleep habits questionnaire among preschool children with autism spectrum disorder. Res Dev Disabil. (2020) 97:103548. 10.1016/j.ridd.2019.103548 31901672

[74] Thorell LB Nyberg L. The childhood executive functioning inventory (CHEXI): a new rating instrument for parents and teachers. Dev Neuropsychol. (2008) 33:536–52. 10.1080/87565640802101516 18568903

[75] Rothbart MK Ahadi SA Hershey KL Fisher P. Investigations of temperament at three to seven years: the children’s behavior questionnaire. Child Dev. (2001) 72:1394–408. 10.1111/1467-8624.00355 11699677

[76] Stelmach-Mardas M Mardas M Walkowiak J Boeing H. Long-term weight status in regainers after weight loss by lifestyle intervention: status and challenges. Proc Nutr Soc. (2014) 73:509–18. 10.1017/S0029665114000718 25192545

[77] Pellegrini M Carletto S Scumaci E Ponzo V Ostacoli L Bo S. The use of self-help strategies in obesity treatment. A narrative review focused on hypnosis and mindfulness. Curr Obes Rep. (2021) 10:351–64. 10.1007/s13679-021-00443-z 34050891 PMC8408071

